# Oncobiguanides: Paracelsus' law and nonconventional routes for administering diabetobiguanides for cancer treatment

**DOI:** 10.18632/oncotarget.1965

**Published:** 2014-05-13

**Authors:** Javier A. Menendez, Rosa Quirantes-Piné, Esther Rodríguez-Gallego, Sílvia Cufí, Bruna Corominas-Faja, Elisabet Cuyàs, Joaquim Bosch-Barrera, Begoña Martin-Castillo, Antonio Segura-Carretero, Jorge Joven

**Affiliations:** ^1^ Metabolism & Cancer Group, Translational Research Laboratory, Catalan Institute of Oncology, Girona, Catalonia (Spain); ^2^ Girona Biomedical Research Institute (IDIBGI), Girona, Catalonia (Spain); ^3^ Department of Analytical Chemistry, Faculty of Sciences,University of Granada, Granada (Spain); ^4^ Unitat de Recerca Biomèdica (URB-CRB), Institut d'Investigació Sanitaria Pere i Virgili (IISPV), Universitat Rovira i Virgili, Reus, Catalonia (Spain); ^5^ Medical Oncology, Catalan Institute of Oncology, Girona, Catalonia (Spain); ^6^ Unit of Clinical Research, Catalan Institute of Oncology, Girona, Catalonia (Spain)

**Keywords:** Metformin, biguanides, diabetes, cancer, insulin, PI3K, PIK3CA mutations

## Abstract

“The dose makes the poison”, the common motto of toxicology first expressed by Paracelsus more than 400 years ago, may effectively serve to guide potential applications for metformin and related biguanides in oncology. While Paracelsus' law for the dose-response effect has been commonly exploited for the use of some anti-cancer drugs at lower doses in non-neoplastic diseases (e.g., methotrexate), the opposite scenario also holds true; in other words, higher doses of non-oncology drugs, such as anti-diabetic biguanides, might exert direct anti-neoplastic effects. Here, we propose that, as for any drug, there is a dose range for biguanides that is without any effect, one corresponding to “diabetobiguanides” with a pharmacological effect (e.g., insulin sensitization in type 2 diabetes, prevention of insulin-dependent carcinogenesis, indirect inhibition of insulin and growth factor-dependent cancer growth) but with minimal toxicity and another corresponding to “oncobiguanides” with pharmacological (i.e., direct and strong anticancer activity against cancer cells) as well as toxic effects. Considering that biguanides demonstrate a better safety profile than most oncology drugs in current use, we should contemplate the possibility of administering biguanides through non-conventional routes (e.g., inhaled for carcinomas of the lung, topical for skin cancers, intravenous as an adjunctive therapy, rectal suppositories for rectal cancer) to unambiguously investigate the therapeutic value of high-dose transient biguanide exposure in cancer. Perhaps then, the oncobiguanides, as we call them here, could be viewed as a mechanistically different type of anti-cancer drugs employed at doses notably higher than those used chronically when functioning as diabetobiguanides.

The antimetabolite drug methotrexate (MTX) was originally developed as an anti-cancer agent and, as such, received U.S. Food and Drug Administration (FDA) approval in 1953. Not long afterwards, MTX was found to exert symptomatic control of severe, recalcitrant, disabling psoriasis; the FDA officially approved MTX as a treatment for psoriasis in the early 1970's. Today, MTX is a well-known chemotherapeutic and immunosuppressive agent that is widely and successfully used in many rheumatologic, dermatologic, and hematologic diseases [1]. Notably, the clinical use of MTX represents a paradigmatic example of Paracelsus' law, which states “*sola dosis facit venenum* (only dose makes the poison)”, meaning that the right dose differentiates a poison from a remedy; hence, a molecule becomes a drug if the dose required to treat a complication is pharmacologically active with minimal toxicity. The so-called Paracelsus' “dose-response effect” establishes that, for any drug, there is a dose range (concentration) that is without any effect, one with a pharmacological effect but minimal toxicity (or an acceptable safety profile), and another with pharmacological and toxic effects. In the case of MTX, experience in multiple sclerosis indicates that the low dose of 7.5 mg per square meter (m^2^) per week (0.1 mg kg^−1^) for up to 2 years is not associated with toxicity. The use of doses of MTX up to 30 mg per week (0.4 mg kg^−1^) in the treatment of juvenile and rheumatoid arthritis and psoriasis is associated with an acceptable toxicity profile. Drastically higher doses of MTX, up to 5,000-12,000 mg per m^2^ (130-300 mg kg^−1^) for several weeks, a dosage that can yield serum concentrations of >1,000 μmol/L (*i.e.,* within the range of concentrations associated with life-threatening MTX toxicity), are used for the treatment of cancer. MTX is therefore used as an “onco drug” at doses up to 1,000-fold higher than those employed chronically for separate indications in immune diseases such as rheumatoid arthritis and multiple sclerosis.

During the early 1970's with the cancer-to-psoriasis drug repositioning of MTX, Canada approved the use of metformin, a member of the biguanide class of drugs that also includes the withdrawn agents phenformin and buformin, for the treatment of type 2 diabetes. Metformin is now one of the most prescribed drugs worldwide; in 2010, there were more than 100 million prescriptions worldwide for metformin, alone and in combination. Starting in 2005 with a report by Evans *et al.* entitled “*Metformin and reduced risk of cancer in diabetic patients*” [2], numerous epidemiological and case-controlled studies have repeatedly suggested that the use of metformin in diabetic patients appears to be associated with a significantly lower risk of cancer mortality and incidence in comparison to other anti-diabetic medications. Since then, the hypothesis that metformin may have clinically relevant preventive and treatment effects against human cancers has exploded as an ever-growing research field, as scientists have discovered metformin's multi-faceted anti-cancer mechanisms linked to malignization and even to so-called cancer stem cells (CSCs) [3-6]. Thus, the molecular and pre-clinical breakthroughs in “anti-cancer metformin” have taken place in the past decade, and many phase II and II clinical trials of metformin are now in progress (as of February 2014, the clinicaltrials.gov database lists more than 60) to examine the effects of metformin on various cancer endpoints.

Although the present, metformin-based clinical investigations aim to test the relevant hypothesis that a practical, long-term administration of conventional anti-diabetic doses of this biguanide may have significant anticancer activity, most of metformin's antineoplastic mechanisms of action that appear to operate in preclinical models [3-6] will remain obscured in these trials, as drug exposure in target cancer tissues may be clearly suboptimal. In the cancer prevention scenario, where side effects are less acceptable than they are in the area of cancer treatment, we might only consider the hypothesis that the conventional dose for the treatment of diabetes, or even lower doses, will be clinically useful. In the cancer treatment scenario, however, the assumption that the antineoplastic effects of metformin are solely attributable to its indirect, endocrine-like effects, such as its insulin-lowering effects, which are generally proposed to slow tumor growth in hyperinsulinemic patients with insulin-addicted cancers, could only explain the currently adopted long-term treatment with diabetic doses for maximal benefit in cancer patients. Conversely, if we assume the real possibility that the antitumoral activity of metformin can be attributed to its many direct actions on target cancer cells as shown *in vitro*, we should then consider that, in certain contexts, short-term exposure to higher doses of biguanides could have clinical use in oncology. Thus, in addition to the fact that particular tissues known to accumulate relatively high metformin levels following conventional oral dosing (*e.g.,* liver, gastrointestinal tract) may provide proof-of-concept clinical models for investigation of the occurrence and relevance of metformin's direct mechanisms of action (*e.g.,* reduction of hepatoma risk, prevention of familial or sporadic intestinal polyposis) [7], we should contemplate the utility of other unconventional routes of short-term high-dose metformin exposure alone and in combination regimens. As for the above-mentioned case of MTX, we here propose that “oncobiguanides” should be viewed as a different type of anti-cancer drugs when employed at doses notably higher than those used chronically when operating as “diabetobiguanides”.

The notion that conventional phase I and II trials must explore the possibility of exposing tumors to the higher biguanide concentrations used in many preclinical models is certainly supported by the strong anti-cancer efficacy of the intraperitoneal high-dose exposure to metformin observed in *PI3K*-mutated, insulin-independent human cancer xenotumors, in which the anti-cancer effects of metformin necessarily involve mechanisms distinct from those that might operate with the doses used in the clinical management of type 2 diabetes [8]. In our hands, *ad libitum* access to water containing oral 250 mg kg^−1^ metformin beginning 1 week prior to inoculation of MCF10DCIS.com breast cancer cells harboring the insulin-unresponsive *PIK3CA*-activating mutation *H1047R* modestly affected the growth of the xenotumors, reaching a maximum of 43% at 4 weeks after cell inoculation and decreasing toward the end of the treatment (approximately 30-35%). Moreover, oral metformin did not affect tumor incidence, the proliferation factor mitotic activity index (MAI), or the anatomopathological features of MCF10DCIS.com cancer tissues. To determine if oral metformin produced plasmatic levels of metformin that could be comparable to those achieved in humans, we recently assessed plasma concentrations of metformin at the end of the 8-week treatment using HPLC coupled to ESI-QTOF-MS. Mice that were treated with an oral dosing schedule achieved a concentration of 4.7 ± 1.3 μmol/L (~0.6 μg/mL) metformin, which is consistent with the steady-state values that have been reported in diabetic patients at the usual clinical doses and schedules. We then measured the circulating insulin level following exposure to oral metformin (even though this is not a diabetic model) and found that oral metformin significantly decreased insulin levels by ~35%. Although it could be hypothesized that a modest but prolonged suppression of insulin signaling by oral metformin at conventional, anti-diabetic doses can be sufficient to inhibit the growth of breast xenotumors, a role for insulin can be eliminated in *PIK3CA H1047R*-mutated MC10DCIS.com cells, which have been shown to proliferate regardless of the presence or absence of insulin *in vitro* and to form tumors that are refractory to dietary restriction (DR) *in vivo*. Therefore, the metformin-induced inhibition of additional signaling pathways, *e.g.,* the liver-derived production of circulating growth factors that ultimately activate local oncogenic signaling in cancer tissues [9] as well as bioenergetic and anabolic machinery in cancer cells, may be involved.

We hypothesized that a different route of administration of metformin, such as intraperitoneal (i.p.) injection, could be more effective because the peak plasma concentrations would be higher than those that could be achieved with oral metformin. Accordingly, insulin-independent, DR-refractory MCF10DCIS.com xenotumors were exquisitely sensitive to the daily, i.p. administration of 200 mg kg^−1^ metformin beginning 1 week before the inoculation of cancer cells. The anti-cancer activity of daily i.p. metformin increased in a time-dependent manner and reached a highly significant >80% inhibition of tumor growth by the end of the 8-week treatment. Moreover, treatment with metformin using the i.p. administration route significantly reduced the proliferation factor MAI by ~50% and notably decreased the tumor cellularity in MCF10DCIS.com cancer tissues [8]. Notably, HPLC-ESI-QTOF-MS pharmacokinetic analysis showed that the plasma levels of metformin immediately after the last i.p. injection were ~150-fold higher than those obtained with the oral dosing schedule. Thus, mice that were treated with the i.p. dosing schedule achieved 679 ± 16 μmol/L (~87 μg/mL) metformin, a circulating dose of metformin that is within the lower limit observed in an individual with metformin poisoning but that was surprisingly well tolerated and did not significantly affect either the general health or the weight of the mice throughout the course of the study. Strikingly, the highly efficacious and extremely high circulating concentrations of metformin achieved following i.p. administration failed to decrease insulin levels to the extent observed with the oral metformin-dosing schedule.

Blood or plasma concentrations of metformin are typically in the range of 7.8-31.2 μmol/L (1-4 μg/mL) in diabetic persons receiving the drug therapeutically; indeed, during controlled clinical trials, the maximum plasma levels did not generally exceed 39 μmol/L (5 μg/mL) metformin, even at maximum doses. Circulating concentrations of 312-836 μmol/L (40-120 μg/mL) can be detected in victims of acute metformin overdose, and concentrations of 624-1,560 μmol/L (80-200 μg/L) are seen in metformin fatalities. Yet, the “unsafe” concentration of metformin is not precisely known. Moreover, the actual relevance of measuring the circulating levels of metformin in predicting the risk of lactic acidosis remains unclear. Additionally, although there is uncertainty concerning the feasibility of administering biguanides through non-conventional routes to investigate the therapeutic value of high-dose transient exposure, we should acknowledge that biguanides have a better safety profile than most oncology drugs in current use [7]. Perhaps it is time to consider that, as with any drug, there is a metformin dose range that is without any effect, one corresponding to “diabetobiguanides” with a pharmacological effect (*e.g.,* insulin sensitization in type 2 diabetes, prevention of insulin-dependent carcinogenesis, indirect inhibition of insulin- and growth factor-dependent cancer growth) but with minimal toxicity and another corresponding to “oncobiguanides” with pharmacological (*i.e.,* direct and strong anti-cancer activity) as well as toxic effects (Fig. 1, top).

**Figure 1:**
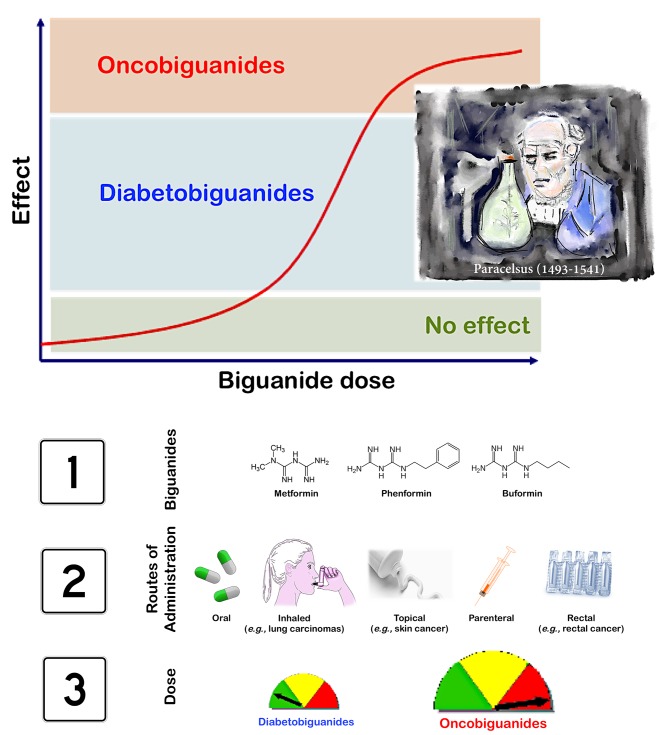
Dose–response anti-cancer effect of biguanides in humans, as per Paracelsus' law *Top panel*. As for any drug, there is a dose range for biguanides that is without any anti-cancer effect, one corresponding to “diabetobiguanides” with a pharmacological effect (*e.g.,* insulin sensitization in type 2 diabetes, prevention of insulin-dependent carcinogenesis, indirect inhibition of insulin- and growth factor-dependent cancer growth) but with minimal toxicity and another corresponding to “oncobiguanides” with pharmacological (*i.e.,* direct and strong activity against cancer cells including CSCs) as well as toxic effects. *Bottom panel*. Because biguanides demonstrate a better safety profile than most oncology drugs in current use, we should contemplate the possibility of administering biguanides through non-conventional routes (*e.g.,* inhaled for carcinomas of the lung, topical for skin cancers, intravenous combination chemotherapy, rectal suppositories for rectal cancer) to unambiguously investigate the therapeutic value of high-dose transient biguanide exposure in cancer. The original painting in the top panel is from Dr. Jorge Joven.

As stated by Paracelsus more than 400 years ago, it is “*the dose that makes a drug*”; this principle has been exploited for the use of some anti-cancer drugs at lower doses in several non-neoplastic diseases. As the opposite scenario also holds true, *i.e.,* higher doses of non-oncology drugs (such as the anti-diabetic biguanides) exerting anti-neoplastic effects, the exploitation of available biguanides (metformin, phenformin, buformin) or libraries of novel biguanides using nonconventional routes of administration (*e.g.,* inhaled, topical, parental, rectal, *etc*) to achieve short-term high-dose exposure in cancer tissues (Fig. 1, bottom) could represent a cost-effective and efficient channel to optimize pharmacokinetics and develop rational combinations in a forthcoming second-generation of biguanide-based trials in oncology. While endless pharmacovigilance has monitored the safety profile of the diabetobiguanide metformin, its natural ancestor, guanidine-rich *Galega officinalis* (known as “Professor-Weed” in the USA), is a Class A Federal Noxious Weed in 35 states of the United States and appears on the database of poisonous plants [10], ironically recapitulating the dose-response effect of metformin in humans, as per Paracelsus' law.
